# Evaluation of Different Nutritional Sources in Lactic Acid Bacteria Fermentation for Sustainable Postbiotic Production

**DOI:** 10.3390/foods14040649

**Published:** 2025-02-14

**Authors:** Chajira Garrote Achou, María J. Cantalejo Díez, Jesús Diaz Cano, Xabier Molinos Equiza

**Affiliations:** 1 Institute for Sustainability & Food Chain Innovation (IS-FOOD), Public University of Navarre (UPNA), Arrosadia Campus, E-31006 Pamplona, Spain; chajira@pentabiol.es; 2 Department of Research and Development, PENTABIOL S.L., E-31191 Esquiroz, Spain; jesus@pentabiol.es (J.D.C.); xabier@pentabiol.es (X.M.E.)

**Keywords:** antibacterial activity, culture media, carbon source, food production, nitrogen source, optimization, plant-based alternatives

## Abstract

In recent years, interest in postbiotics has grown due to their potential health benefits and applications in food systems. This study evaluated various nutritional sources for lactic acid bacteria (LAB) fermentation to enhance postbiotic production. Three LAB strains were tested: *Pediococcus acidilactici* CECT 9879 (PA), *Weissella cibaria* CECT 30731 (WC), and *Lactococcus lactis* CECT 30734 (LL). Fermentation experiments assessed bacterial growth, pH levels, and antibacterial activity against *E. coli* using different carbon and nitrogen sources. Fructose and xylose significantly improved growth in WC (9.39 ± 0.16 log CFU/mL) and LL (9.37 ± 0.22 log CFU/mL) compared to glucose. Ribose enhanced antimicrobial activity in PA (41.67 ± 2.89%) and WC (50.00 ± 0.00%) relative to glucose. Additionally, plant-based nitrogen sources, such as soy (LL: 8.93 ± 0.12 log CFU/mL and 81.67 ± 2.89%) and wheat (WC: 9.40 ± 0.17 log CFU/mL and 65.00 ± 0.00%), along with microbial sources like yeast (PA: 9.57 ± 0.12 log CFU/mL and 40.00 ± 0.00%), effectively supported growth and antibacterial activity. These findings highlight the potential of developing animal-free fermentation media that meet nutritional, safety, and sustainability criteria while making a significant contribution to the optimization of postbiotic production.

## 1. Introduction

Lactic acid bacteria (LAB) are essential microorganisms in fermentation processes, contributing to the production of a variety of metabolic products that add significant value to the food industry. LAB fermentation processes are commonly carried out in either solid-state (SSF) or liquid-state (SLF) systems, with liquid-state fermentation (SLF) being the most widely studied and industrially implemented. During fermentation, LAB secrete metabolites into the surrounding environment, primarily through the supernatant, which may include organic acids like lactic acid, antimicrobial peptides, and other bioactive compounds. In addition to these by-products, the biomass generated during fermentation can also have probiotic or postbiotic potential [1]. Due to their metabolic characteristics, LAB are used not only to enhance the flavor and nutritional value of fermented foods and extend shelf life, but also to inhibit pathogenic bacteria and promote health [2]. This broad range of applications underscores the importance of optimizing LAB fermentation processes, laying the foundation for further advancement in microbial product formulations.

Building upon these applications, recent research has also focused on the properties of inactivated bacterial products. Postbiotics, for instance, are characterized by several intriguing features, including a unique chemical composition, safety, ease of storage, user-friendliness, stability across a wide range of pH levels and temperatures, and broad-spectrum antimicrobial properties [3]. For example, Yavorov-Dayliev et al. [4] studied strain CECT 9879 in its postbiotic form. Their findings demonstrated that this form significantly reduced fasting blood glucose levels and insulin resistance during gestation, while also exhibiting lower visceral adiposity, increased muscle tissue, and improvements in intrahepatic triglyceride content and ALT levels. In addition, postbiotic supplementation modulated gut microbiota composition in mice. Similarly, strain CECT 30731, in its postbiotic form, exhibited immunomodulatory effects, enhanced intestinal microbial composition, and improved the resistance of rainbow trout (*Oncorhynchus mykiss*) against *Yersinia ruckeri* [5]. These promising results underscore the potential for optimizing inactivated bacterial products as a part of advanced microbial applications in the health and food sectors.

At the industrial level, several important factors must be considered to maximize the yields of desired products during fermentation, such as the choice of culture medium. The current market offers a wide range of commercial growth media for lactic acid bacteria, with the most commonly used being Man Rogosa and Sharpe (MRS), Brain Heart Infusion (BHI), and Tryptone Glucose Extract (TGE), among others [6]. The use of these media promotes appropriate growth and enhances the synthesis of desirable products. However, since these media are designed for the growth of a broad spectrum of bacteria, their composition and concentration may not be optimal for specific strains. Limitations arise from factors such as the lack of essential molecules required for cellular metabolism, nutrient deficiencies during the exponential phase, and insufficient sources of carbon and nitrogen preferred by some strains [7].

Optimizing fermentation processes is critical for enhancing microbial growth and the production of valuable metabolites. A key factor in achieving this is the optimization of growth media. One widely used approach is the one-factor-at-a-time (OFAT) method, which involves varying individual factors while keeping others constant until an apparent optimal condition is reached. It is a useful method for identifying the primary factor responsible for the observed effect [8]. This simple and convenient method is particularly used in the early stages of medium formulation [9]. Although significant research efforts have focused on optimizing probiotic production [10,11,12,13] and purified products of bacterial fermentation—such as lactic acid [14,15,16], exopolysaccharides (EPSs) [17,18,19], or gamma-aminobutyric acid (GABA) [20,21]—investigations into the fermentation conditions that affect postbiotic production remain limited.

Building on the importance of optimizing growth media, there is a parallel shift towards sustainable alternatives in food production. Recently, the move to replace animal-sourced ingredients with plant-based alternatives (PBAs) has gained momentum as a solution to reduce environmental impact, improve health, and enhance animal welfare and food security. Diets rich in PBAs have been shown to significantly lower greenhouse gas emissions, land use, and water consumption [22]. In Western Europe, it is increasingly recognized that adopting more plant-based diets is crucial to achieving both global food system sustainability and better public health [23]. This transition extends beyond dietary choices to industrial and biotechnological processes, such as microbial culture production. Traditionally, the media used to grow microorganisms, including lactic acid bacteria (LAB), contain animal-derived components, such as meat extracts and peptones. While these animal-based ingredients are effective for microbial growth, they pose contamination risks [24], particularly with the emergence of transmissible prion diseases, such as bovine spongiform encephalopathy (BSE) [25]. Furthermore, the use of animal-derived materials may conflict with dietary preferences and cultural standards, including vegan, vegetarian, and halal requirements [26,27]. Developing animal-free culture media can mitigate contamination risks and align with broader sustainability goals. However, it is crucial to ensure that these alternatives provide the necessary nutrients for optimal growth and the production of metabolic products. Complete substitution presents challenges, as animal-derived components perform critical functions in the media. Furthermore, the available literature provides limited insight into using plant-based substrates to support LAB growth [28], particularly in postbiotic production.

In response to the growing demand for sustainable and ethical practices in food production, this study explores various nutritional sources to optimize the composition of a fermentation culture medium. The formulated medium is then used to assess the growth, antimicrobial activity, and final fermentation pH of three lactic acid bacteria (LAB) strains.

## 2. Materials and Methods

### 2.1. Experimental Design

**Figure 1:**
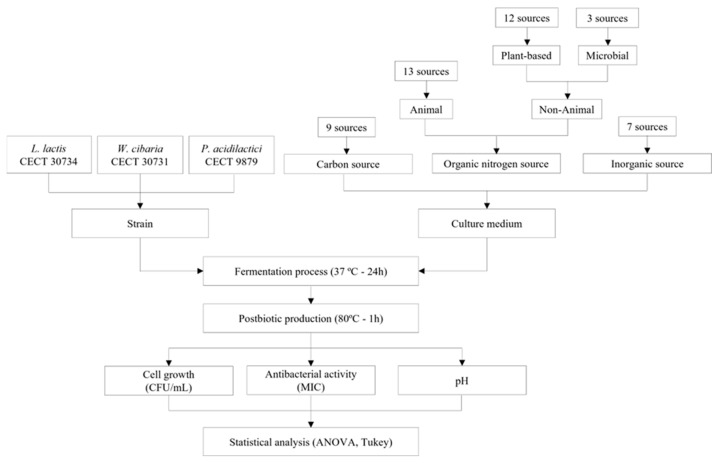
Experimental design for the evaluation of different nutritional sources in lactic acid bacteria fermentation for sustainable postbiotic production.

The methodology employed in this study is illustrated in Figure 1. The experiments were designed to include medium preparation, inoculation with selected strains, fermentation process, and measurement of response variables. Based on identifying optimal conditions to maximize production and to explore alternative sources to those of animal origin commonly used in commercial MRS media, the figure represents the key steps of the experimental process, providing a structured overview of the approach followed.

### 2.2. Bacterial Strains and Culture Conditions

The LAB strains used in this study, *P. acidilactici* CECT 9879, *L. lactis* CECT 30734, and *W. cibaria* CECT 30731, were provided by the Spanish Type Culture Collection (CECT, Paterna, Spain). These strains were selected based on the proven postbiotic effects documented in the literature [4,5,29]. Furthermore, they were evaluated by PENTABIOL S.L. (Esquíroz, Spain) in the development of their registered trademark Probisan^®^ products [30], making them suitable for optimizing studies. The lyophilized strains were reconstituted in 0.2 mL of Man Rogosa Sharpe broth (MRS, Condalab, Laboratorios Conda S.A., Madrid, Spain). Subsequently, the solution was introduced into 5 mL of MRS broth and incubated (Lan Technics, Model 509308, Esparza de Galar, Spain) for 24 h at 37 °C. A second subculturing was conducted by adding 1 mL of the previous microbial culture to 9 mL of MRS broth. Glycerol (30% *v*/*v*) was then added, and the bacteria were stored at −80 °C for subsequent experiments. This study utilized *E. coli* (strain number: 097040) as an indicator strain, isolated from a clinical case in young goats on a livestock farm in Spain, obtained from the microorganism collection of PENTABIOL S.L. The strain was stored at −80 °C in Trypticase Soy Broth (TSB, Condalab) with glycerol (30% *v*/*v*). Prior to the experiment, the strain underwent two subcultures at 37 °C for 24 h.

### 2.3. Fermentation and Inactivation Process

The fermentation process for each experimental medium followed a standardized procedure. First, 50 µL of the stock culture was reactivated in 5 mL of the respective experimental medium broth and incubated at 37 °C for 24 h. Then, 1 mL of the seed culture was used to inoculate 9 mL of the experimental medium, which was incubated at 37 °C for an additional 24 h without agitation. For each experimental medium, a basal medium based on the MRS formula was prepared, consisting of 5 g/L sodium acetate, 2 g/L K_2_HPO_4_, 1 g/L Tween 80, 0.2 g/L MgSO_4_·7H_2_O, and 0.05 g/L MnSO_4_·H_2_O (Barcelonesa, Barcelona, Spain). The MRS medium, prepared by supplementing the basal medium with 20 g/L glucose, 10 g/L bacteriological peptone (Condalab), 8 g/L meat extract (Hispanagar S.A., Burgos, Spain), and 4 g/L yeast extract (Hispanagar), was used as the control for comparison with the experimental media. Inactivated bacterial cultures were produced by subjecting the experimental cultures to thermal treatment at 80 °C for 1 h using an autoclave (Daihan Scientific, Maxterile 60, Gangwon-do, Republic of Korea). To confirm the absence of viable bacteria, 100 µL of the final inactivated culture was spread onto MRS agar plates and incubated at 37 °C for 48 h. The absence of visible growth indicated the successful inactivation process of the bacterial fermentation [29].

### 2.4. Measurement of Cell Growth and pH Levels

After the fermentation process, serial decimal dilutions were prepared, and the number of cells was detected using the plate count method with MRS agar. The plates were incubated at 37 °C for 48 h, and the bacterial count was expressed as Log CFU/mL. A pH meter (HI5521; Hanna Instruments, Woonsocket, RI, USA) connected to an electrode (HI1131; Hanna Instruments) was used to measure the acidity of the media. Each parameter for all strains was tested in triplicate.

### 2.5. Determination of Antibacterial Activity

The antibacterial activity of the cell-free inactivated supernatants against *E. coli* was evaluated using the Minimum Inhibitory Concentration (MIC) assay in 96-well plates, following the protocol by Pelyuntha et al. [31] with minor modifications. Inactivated bacterial cultures were centrifuged (Lan Technics, DM0412) at 2400× *g* for 10 min. The supernatants were collected and serially diluted in Brain Heart Infusion broth (BHI, Condalab) to achieve final concentrations ranging from 5% to 100% (*v*/*v*). Then, 150 μL of the indicator strain adjusted to 10^5^ CFU/mL was added to each well. Positive and negative controls were incubated separately in different wells of the same plates. The positive control contained the pathogen in BHI, while the negative control contained the cell-free inactivated supernatants in BHI without the pathogen. The plates were covered and incubated at 37 °C for 24 h under aerobic conditions before reading. The MIC value was defined as the lowest concentration of cell-free inactivated supernatants that showed no macroscopically visible growth.

### 2.6. Effect of Carbon Sources

To investigate the effect of different carbon sources on the fermentation culture medium, nine carbon sources were individually added to the basal medium at a concentration of 20 g/L. These sources included glucose (Pintaluba S.A., Tarragona, Spain), fructose (TCI, Tokyo Chemical Industry Co., Ltd., Tokyo, Japan), xylose (Laboratoriumdiscounter, Ijmuiden, The Netherlands), galactose (ROTH, Carl Roth GmbH + Co KG, Karlsruhe, Germany), ribose (TCI), maltose (TCI), trehalose (TCI), lactose (TCI), and starch (VWR International, Leuven, Belgium). A fixed nitrogen source, consisting of 10 g/L bacteriological peptone, 8 g/L meat extract, and 4 g/L yeast extract, was used for all media. The selection of these carbon sources was based on the carbohydrate fermentation patterns observed in the API 50 CH test, which was performed following the manufacturer’s instructions. The results were recorded after incubation at 37 °C for 48 h.

### 2.7. Effect of Organic Nitrogen Sources of Animal Origin

To evaluate the impact of various animal-derived nitrogen sources on the fermentation culture medium, thirteen nitrogen sources were individually tested: acid casein peptone (Hispanagar), casein peptone (Condalab), proteose peptone No. 3 (Hispanagar), peptonized milk (Kerry, Norwich, NY, USA), meat extract (Hispanagar), tryptone (Condalab), gelatin peptone (Condalab), bacteriological peptone (Condalab), lactalbumin hydrolysate (Hispanagar), acid casein hydrolysate (Kerry), enzymatic casein hydrolysate (Kerry), bacteriological peptone type II (Hispanagar), and casein peptone type IV (Hispanagar). These sources were added to the basal medium at a concentration of 22 g/L to create the experimental nitrogen media of animal origin, while a fixed carbon source of 20 g/L glucose was used.

### 2.8. Effect of Organic Nitrogen Sources of Non-Animal Origin

The impact of various plant-based nitrogen sources on fermentation culture medium was evaluated by individually testing twelve nitrogen sources: pea hydrolysate 1.1 (Kerry), rice hydrolysate (Kerry), soy peptone (Hispanagar), wheat peptone (Hispanagar), malt extract (Condalab), acid soy hydrolysate (Kerry), enzymatic soy hydrolysate (Kerry), ultra-filtered cotton hydrolysate (Kerry), soy peptone type III (Hispanagar), pea hydrolysate 3.0 (Kerry), ultra-filtered wheat hydrolysate (Kerry), and ultra-filtered rice hydrolysate (Kerry). These nitrogen sources were added to the basal medium at a concentration of 22 g/L to create the various experimental nitrogen media of plant origin, with a fixed carbon source of 20 g/L glucose. To assess the impact of microbial-derived nitrogen sources on the fermentation process, three distinct yeast extracts were individually tested: yeast extract 5.5 (Kerry), yeast extract 5.4 (Hispanagar), and yeast extract 1.1 (Kerry). Each of these yeast extracts was incorporated into the basal medium at a concentration of 22 g/L to formulate the different experimental nitrogen media of microbial origin, also with a fixed carbon source of 20 g/L glucose.

### 2.9. Effect of Inorganic Sources

To investigate the effect of various inorganic sources on the fermentation culture medium, eight sources were examined: ammonium sulfate (Supelco^®^, Sigma-Aldrich, Darmstadt, Germany), ammonium chloride (Supelco^®^), ammonium citrate (ROTH), potassium nitrate, sodium nitrate, sodium citrate, and sodium sulfate (Laboratoriumdiscounter). These sources were incorporated into the basal medium at a concentration of 22 g/L to create experimental inorganic media, with a fixed carbon source of 20 g/L glucose.

### 2.10. Statistical Analysis

All experiments were conducted in triplicate, and the data are presented as the mean ± standard deviation (SD). Statistical analysis was performed using one-way analysis of variance (ANOVA) followed by Tukey’s post hoc test to compare results. Differences between means were considered significant at *p* ≤ 0.05. All statistical analyses were conducted using Minitab^®^ Statistical Software Version 22 (Minitab LLC, State College, PA, USA).

## 3. Results and Discussion

### 3.1. Effect of Carbon Sources on Bacterial Growth, pH, and Antibacterial Activity

According to the data in Table 1, one of the highest bacterial concentrations for the PA strain was observed with fructose, with no significant difference compared to glucose, the carbon source in the MRS formulation. The lowest concentration for PA was recorded with maltose. For the WC strain, fructose resulted in the highest bacterial concentration, significantly exceeding all other carbon sources, while xylose and maltose produced the lowest concentrations. In the LL strain, the highest bacterial concentrations were achieved with xylose and starch, significantly surpassing all other tested carbon sources, while fructose resulted in the lowest concentration for this strain. The observed differences in bacterial concentration with the various sugars may reflect the metabolic preferences and specific capabilities of each strain to assimilate and utilize these carbohydrates, emphasizing the need to tailor nutrient formulations to the distinct metabolic requirements of each strain to optimize bacterial yield. Similar observations were reported by Fuso et al. [32], who demonstrated that the growth dynamics of various *Lactobacillus* strains were highly dependent on the sugar source provided. Notably, *L. bulgaricus* 1932 exhibited relatively consistent growth across different sugars, whereas *L. rhamnosus* 1019 and *L. paracasei* 2333 showed pronounced variations in growth based on the sugar source. Specifically, *L. rhamnosus* 1019 exhibited reduced growth capacity when maltose and sucrose were used as carbon sources.

The data in Table 1 show that fructose resulted in one of the lowest pH values for strains PA and WC, which is consistent with the high bacterial concentrations observed. For strain LL, glucose led to the lowest pH, but also to one of the lowest bacterial concentrations, likely due to rapid acid accumulation that inhibits further growth. These findings highlight the important role of pH during fermentation in regulating bacterial growth. PA and WC appear to be more tolerant to acidic conditions, efficiently utilizing fructose for both acid production and bacterial growth. In contrast, strain LL shows lower bacterial concentrations under low pH conditions with glucose, and higher concentrations under higher pH conditions with starch, suggesting that LL is more sensitive to the acidic environment it generates. This sensitivity is a characteristic commonly observed in lactic acid bacteria (LAB) [33].

**Figure 2:**
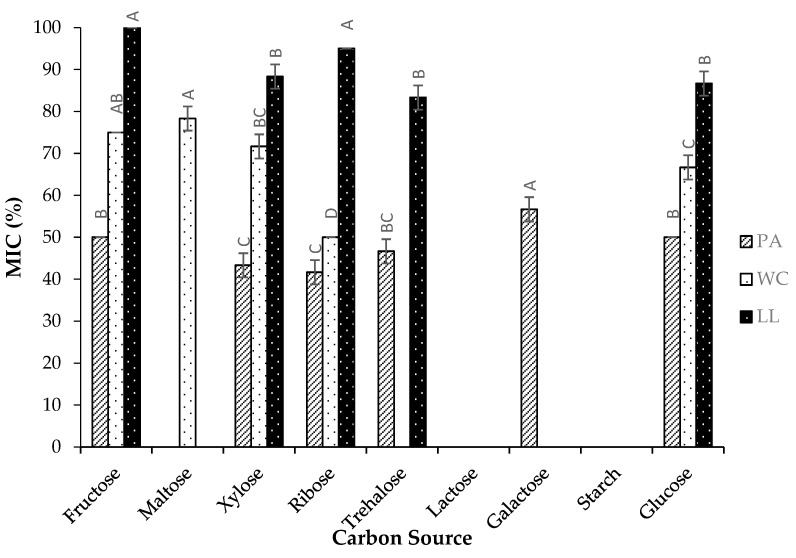
Effect of different carbon sources on the Minimum Inhibitory Concentration (MIC) of three LAB strains (% mean ± SD) against *E. coli*: *Pediococcus acidilactici* (PA), *Weissella cibaria* (WC), and *Lactococcus lactis* (LL). For each strain, values with different uppercase letters are significantly different between carbon sources (*p* ≤ 0.05).

The results presented in Figure 2 reveal significant variations in the Minimum Inhibitory Concentration (MIC) among different carbon sources for strains PA, WC, and LL. In strain PA, the consumption of xylose and ribose resulted in significantly lower MIC values compared to glucose, indicating that these carbon sources enhance antimicrobial production. Conversely, the use of galactose led to the highest MIC, suggesting a reduced effectiveness in this strain. For strain WC, ribose was the most effective carbon source, resulting in the lowest MIC, while maltose resulted in one of the highest MICs. In strain LL, trehalose, glucose, and xylose resulted in equivalent MIC values, indicating similar antimicrobial activity. The highest MIC values in strain LL were observed with fructose and ribose. Although bacterial growth was observed with all tested carbon sources, neither lactose nor starch demonstrated antimicrobial activity across any strain. Notably, galactose showed antibacterial activity only in strain PA, maltose was effective only in strain WC, and trehalose did not exhibit antimicrobial activity in strain WC. The observed variations in MIC values can be attributed to the efficiency of carbohydrate metabolism, which is crucial for antimicrobial production. Lactic acid bacteria (LAB) exhibit varying abilities to metabolize different carbon sources, largely due to the specific activities of the enzymes involved in carbohydrate breakdown. LAB typically have complex enzymatic systems that allow them to metabolize a wide range of carbohydrates. However, certain carbohydrates, such as starch, can negatively influence bacteriocin production, potentially due to interactions where bacterial cells attach to starch surfaces, limiting the efficient use of these carbohydrates. While many bacteria efficiently ferment simple sugars like monosaccharides and disaccharides, only a few are equipped to break down complex carbohydrates such as starch [34].

### 3.2. Effect of Organic Nitrogen Sources of Animal Origin on Bacterial Growth, pH, and Antibacterial Activity

As illustrated in Table 2, one of the highest bacterial concentrations for strains PA and WC was observed with lactalbumin hydrolysate and bacteriological peptone, respectively. These concentrations were significantly higher compared to those achieved with the nitrogen sources used in the MRS control. In the case of strain LL, no significant differences were observed among the tested animal-derived nitrogen sources, except for acid casein peptone and acid casein hydrolysate, which resulted in the lowest growth levels. This suggests that the remaining nitrogen sources are equally effective in supporting the growth of this strain. Across all strains, nitrogen sources derived from acid hydrolysis consistently resulted in the lowest bacterial concentrations, likely due to the degradation of essential nutrients during the acid hydrolysis process [35].

LAB exhibit a rapid intracellular metabolism and require many free amino acids for their growth. Since milk does not contain sufficient free amino acids, these bacteria break down proteins, primarily casein, to obtain the necessary nutrients. The extracellular proteases (CEPs) of LAB initiate the degradation of casein, which is classified into αs1-, αs2-, β-, and κ-casein. These proteases preferentially act on the hydrophobic parts of casein and degrade it into smaller peptides. Despite the high structural similarity, lactic acid bacterial proteins show differences in substrate specificity. The peptides formed are transported into the cell and further broken down by endopeptidases and aminopeptidases, generating di- and tripeptides. Enzymes such as PepN, PepX, and PepA have different specificities for the peptides, and their action releases free amino acids, which are essential for protein synthesis and LAB growth, thereby reducing the energy expenditure on de novo amino acid synthesis [36]. The differences in growth observed among the strains with various peptone sources can be explained by the unique way each bacterial strain responds to these sources. This variation is likely due to the specific enzymes each strain uses to break down and utilize the peptides in the different peptones.

The data revealed significant variations in pH values among the nitrogen sources tested for strains PA, WC, and LL. The lowest pH values were observed with bacteriological peptone type II for strain PA, meat extract and gelatin peptone for strain WC, and peptonized milk for strain LL, suggesting that these sources were more efficiently utilized for acid production, leading to greater acidification of the medium (Table 2). Conversely, acid casein peptone consistently produced higher pH values across all strains, indicating reduced acid production and less effective metabolic utilization for this specific purpose. These findings align with previous studies, such as those by Atilola et al. [37], who observed final pH values ranging from 4.51 to 5.30 when testing various nitrogen sources like proteose peptone, tryptone, and beef extract with four strains of *L. reuteri*. Although carbohydrates are the primary contributors to acidification and pH fluctuations during fermentation, as they are converted into organic acids by lactic acid bacteria [38], the nitrogen source also plays an important role. The ability of bacteria to effectively metabolize specific nitrogen sources can influence acid production by supporting key metabolic processes. For instance, Mis Solval et al. [39] noted that the pH of fermentation media during the cultivation of *L. plantarum* NRRL B-4496 was significantly lower in MRS and MRSN-EWH (MRS with dried egg white hydrolysates as the nitrogen source) compared to MRSN (MRS without a nitrogen source). This finding emphasizes the interaction between carbohydrate and nitrogen sources in regulating pH dynamics during fermentation.

**Figure 3:**
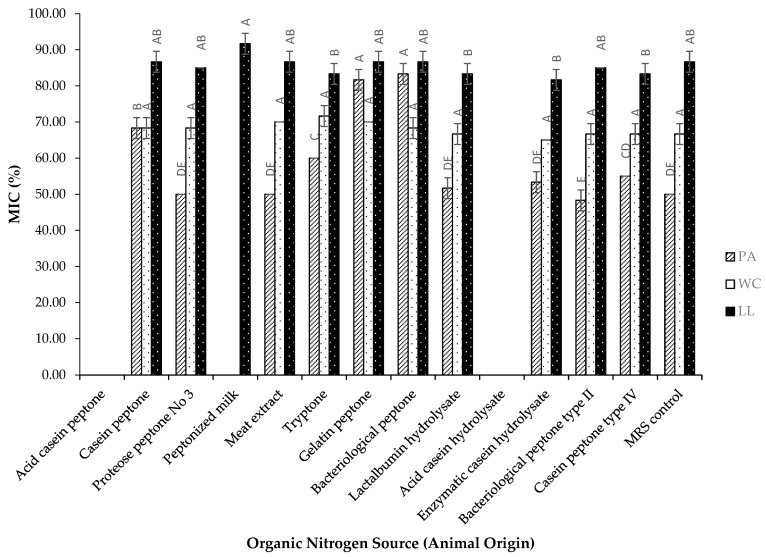
Effect of different nitrogen sources of animal origin on the Minimum Inhibitory Concentration (MIC) of three LAB strains (% mean ± SD) against *E. coli*: *Pediococcus acidilactici* (PA), *Weissella cibaria* (WC), and *Lactococcus lactis* (LL). For each strain, values with different uppercase letters are significantly different between carbon sources (*p* ≤ 0.05).

The data presented in Figure 3 indicate that for the PA strain, the MIC values were notably influenced by changes in nitrogen sources. One of the lowest MIC values was observed with bacteriological peptone type II, which was comparable to the MRS control. In contrast, the highest MIC values were associated with bacteriological peptone and gelatin peptone. For the WC strain, no statistically significant differences were found among the various animal-origin nitrogen sources. Meanwhile, the LL strain exhibited minimal variation in MIC values across the different nitrogen sources. Notably, when the PA and WC strains were cultured with peptonized milk, no antibacterial activity was detected. Additionally, nitrogen sources produced by acid hydrolysis did not result in any antibacterial activity for any of the three strains. The findings of this study reveal that the conditions favorable to antibacterial activity production did not always coincide with those that promoted optimal bacterial growth. For instance, in the PA strain, bacteriological peptone and gelatin peptone supported substantial bacterial growth but exhibited limited inhibitory activity. In contrast, lactoalbumin hydrolysate not only facilitated robust growth but also demonstrated strong antibacterial effects, as reflected by one of the lowest MIC values. These observations align with the findings of Aguilar-Galvez et al. [40], who reported that in the *E. faecium* CWBI-B1430 strain, beef extract led to the highest anti-*Listeria* activity, while soy peptone produced the maximum biomass. This reflects the principle that bacteriocin production in lactic acid bacteria is often linked to growth kinetics, typically peaking during the exponential phase and diminishing in the stationary phase. However, a high bacterial biomass does not always equate to increased bacteriocin activity, which may be limited by low specific production rates per cell mass. This underscores the complex and strain-specific interplay between environmental conditions, bacterial growth, and bacteriocin production [34].

### 3.3. Effect of Organic Nitrogen Sources of Non-Animal Origin on Bacterial Growth, pH, and Antibacterial Activity

When soy peptone was used in the culture medium, the PA and LL strains showed some of the highest bacterial concentrations, with values statistically comparable to those obtained from other plant-based sources. For the PA strain, the concentrations were similar to those achieved with the MRS control, while for the LL strain, the concentrations were significantly higher than those observed with MRS (Table 3). This suggests that soy peptone can effectively support the growth of these strains, potentially offering a viable alternative to traditional animal nitrogen sources. For the WC strain, one of the highest bacterial concentrations was recorded with ultra-filtered wheat hydrolysate, significantly surpassing the concentration observed in MRS and further underscoring the potential of plant-based substrates in LAB cultivation. This finding is particularly important given the growing interest in sustainable and safe alternatives for microbial cultivation [24,26,41].

The bacterial concentrations of different yeast extracts were analyzed across the three strains. For the PA strain, the lowest concentration was recorded with yeast extract 1.1, while the concentrations obtained from the other yeast extracts were comparable to those of the MRS control. For the WC and LL strains, no significant differences were observed between the yeast extracts and the MRS control, as detailed in Table 3. These findings underscore the nutritional richness of yeast extract, which offers essential growth factors for lactic acid bacteria, including nucleic acids, proteins, and B vitamins [42]. Components such as glucans, mannans, chitin, and other macromolecules from yeast extract provide a more balanced nutritional profile compared to plant-based supplements. Additionally, ribose, the primary reducing sugar found in yeast extract, serves as a key precursor in cellular energy metabolism [43]. Previous studies on essential nutritional supplements for the growth of *Lactobacillus johnsonii* NRRL B-2178 found that cell growth is directly related to the concentration of yeast extract. It was also discussed that the required amount of yeast extract depends on its nucleotide content, which can vary depending on the supplier [44].

The data presented in Table 3 indicate that the type of nitrogen source significantly affects pH variation during fermentation, highlighting differences in acid production among the strains. For both the PA and LL strains, enzymatic soy hydrolysate resulted in notably lower pH values, suggesting greater acid production compared to the other plant-based nitrogen sources. In the case of the WC strain, soy peptone produced the lowest pH value, demonstrating its effectiveness in enhancing acid production. These findings are consistent with those reported by Atilola et al. [37], who observed similar pH values (4.51 to 5.02) using a papain digest of soybean meal as a nitrogen source for four *L. reuteri* strains, further supporting the efficiency of soy-based hydrolysates. In contrast, the highest pH values were observed in rice hydrolysate for the PA and WC strains and with acid soy hydrolysate for the LL strain, indicating reduced acid production.

Similarly, significant differences were observed when testing yeast extracts as nitrogen sources. For the PA strain, yeast extract 5.5 led to the lowest pH, reflecting greater acid production, while yeast extract 1.1 resulted in the highest pH. In contrast, for the WC strain, yeast extract 5.4 produced the lowest pH, while yeast extract 5.5 yielded the highest. For the LL strain, yeast extract 1.1 was associated with the lowest pH, whereas yeast extract 5.4 generated the highest pH. These variations in pH among nitrogen sources underscore the influence of nitrogen availability and composition on strain-specific metabolic activity, further supporting the idea that certain nitrogen sources better support the biosynthetic pathways involved in acid production.

**Figure 4:**
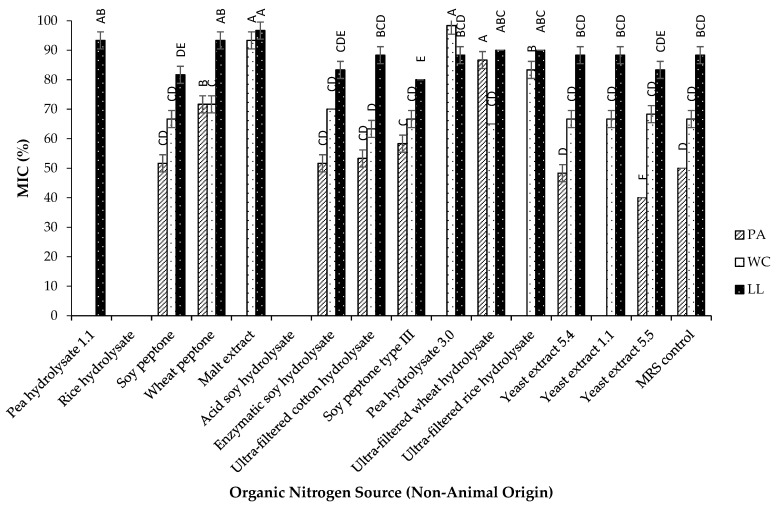
Effect of different nitrogen sources of non-animal origin on the Minimum Inhibitory Concentration (MIC) of three LAB strains (% mean ± SD) against *E. coli*: *Pediococcus acidilactici* (PA), *Weissella cibaria* (WC), and *Lactococcus lactis* (LL). For each strain, values with different uppercase letters are significantly different between carbon sources (*p* ≤ 0.05).

The results presented in Figure 4 show that the PA strain exhibited MIC values for certain plant-based nitrogen sources, such as those derived from soy and cotton, that were statistically similar to the values obtained with MRS. In contrast, ultra-filtered wheat hydrolysate resulted in significantly higher MIC values than the other tested nitrogen sources. For the WC strain, nitrogen sources from cotton, wheat, and soy also showed no significant difference in MIC values compared to MRS. The highest MIC values for this strain were recorded with pea hydrolysate 3.0 and malt extract. For the LL strain, nitrogen sources from soy, cotton, wheat, rice, and pea produced MIC values similar to those of MRS. However, poor performance was observed with malt extract, which resulted in some of the highest MIC values. These findings are consistent with certain reports in the literature, where soy peptone was found to enhance the bacteriocin activity of *Pediococcus pentosaceus* [45] and *Lactococcus lactis* [46], highlighting its potential as an effective nitrogen source.

In a similar manner to animal-derived nitrogen sources, the nitrogen sources obtained through acid hydrolysis did not demonstrate any antibacterial activity in any of the three strains. The PA strain exhibited a high sensitivity to variations in plant-based nitrogen sources, showing a loss of antibacterial activity when exposed to pea hydrolysate 1.1, rice hydrolysate, malt extract, pea hydrolysate 3.0, and ultra-filtered rice hydrolysate. Meanwhile, the WC strain completely lost its antibacterial activity when exposed to pea hydrolysate 1.1 and rice hydrolysate. The LL strain, however, demonstrated greater adaptability, as only the rice hydrolysate resulted in a complete loss of activity. These results indicate that the PA strain is particularly sensitive to changes in nitrogen composition, especially from plant-based sources, while the LL strain exhibits greater flexibility in utilizing diverse nitrogen sources. While legumes and cereals are abundant in macronutrients and micronutrients, they may also contain anti-nutritional factors [47]. For instance, although peas are high in protein, their efficacy can be diminished by anti-nutritional components such as tannins and trypsin inhibitors. Additionally, oligosaccharides like raffinose, stachyose, and verbascose can further impair nutrient digestibility [48]. These findings highlight the challenges associated with replacing animal-derived nitrogen sources with plant-based alternatives. While all three strains showed inhibition against most of the animal-derived nitrogen sources tested, the variability in their response to plant-based sources reflects the complexity of this substitution. This emphasizes the need for careful selection and optimization of plant-based nitrogen sources to maintain or enhance antibacterial activity, particularly in strains that are more sensitive, such as PA.

Analysis of Figure 4 reveals that the PA strain experienced a decline in antibacterial activity when yeast extract 1.1 was utilized as the nitrogen source. Variations in yeast extract composition, influenced by differences in raw materials and production processes, make it crucial to select the appropriate extract for each microorganism to optimize fermentation [43]. In contrast, the PA strain demonstrated its lowest MIC value with yeast extract 5.5, significantly lower than those observed with yeast extract 5.4 and MRS. For the WC and LL strains, no significant differences were observed among the yeast extracts tested, with all extracts yielding results comparable to the MRS control. Yeast extract is well-known for its effectiveness in enhancing bacteriocin production in various lactic acid bacteria (LAB). Studies have confirmed that yeast extract supplementation in culture media notably increases bacteriocin production in *Lactococcus lactis* subsp. *lactis* [49], *Lactobacillus acidophilus* [50], and *Lactobacillus paracasei* subsp. *tolerans* [51].

While this study primarily focuses on optimizing fermentation media and its impact on postbiotic production, it is important to recognize the potential applications of these findings in food systems, particularly in the field of biopreservation. Incorporating postbiotic strains into food products not only enhances their functional value but also plays a key role in food preservation. Postbiotics present a variety of antimicrobial substances that help reduce pathogen loads and neutralize harmful toxins from foodborne pathogens such as *Salmonella* spp., *Escherichia coli*, *Listeria monocytogenes*, and fungi [52].

### 3.4. Effect of Inorganic Sources on Bacterial Growth, pH, and Antibacterial Activity

For the PA strain, growth was observed exclusively in the presence of ammonium sulfate, ammonium chloride, ammonium citrate, and sodium nitrate. The highest bacterial concentration was achieved with ammonium sulfate, which was significantly higher compared to the other inorganic sources tested. The lowest concentration was recorded with sodium nitrate. The WC strain exhibited growth across all tested inorganic sources, with the highest concentration observed when potassium nitrate was used. This value, however, was not significantly different from those obtained from most other sources, except for ammonium chloride and sodium sulfate. Among these, sodium sulfate resulted in the lowest bacterial concentration for this strain. The LL strain only grew in the presence of ammonium sulfate, ammonium chloride, and ammonium citrate, with ammonium sulfate leading to a significantly higher concentration compared to the other sources. The lowest concentration for LL was recorded with ammonium citrate, as shown in Table 4.

The results obtained in this study demonstrate that the bacterial strains exhibited significantly better growth when cultured in MRS medium, which contains organic nitrogen sources, compared to the inorganic nitrogen sources tested. This difference can be explained by the fact that organic nitrogen, composed of peptides and free amino acids, is readily absorbed by bacterial cells from the medium. Consequently, these complex nitrogen sources are directly incorporated into proteins or transformed into other nitrogenous cellular components. In contrast, utilizing inorganic nitrogen sources requires the cells to expend more energy and time to synthesize amino acids for protein synthesis [34], imposing an additional metabolic burden that likely hampers their growth, as reflected by the lower bacterial concentrations observed.

Regarding pH, none of the tested strains exhibited a decrease below 5.75 when grown with inorganic nitrogen sources, and no antibacterial activity was detected under these conditions. This finding aligns with the results reported by Jawan et al. [46], who found that substituting organic nitrogen sources with inorganic alternatives generally results in reduced antibacterial activity. Furthermore, the inability of certain strains to grow in specific inorganic nitrogen media emphasizes the essential role of organic nitrogen sources in optimizing bacterial growth and metabolite production.

## 4. Conclusions

The outcomes of this research provide valuable insights into the complex dynamics of nutrient sources in lactic acid bacteria fermentation. The data reveal that the use of fructose and xylose significantly enhances bacterial growth in the WC and LL strains, respectively, while ribose improves antimicrobial activity in the PA and WC strains compared to the commonly used glucose in the commercial MRS medium. The results demonstrate that the conditions favoring bacterial growth do not always align with those enhancing antibacterial activity. Although all tested carbon and organic nitrogen sources supported growth across all strains, antimicrobial activity varied significantly depending on the specific source used. Since each nitrogen source contains different peptides and amino acids, strain-specific responses were observed, making it essential to evaluate the most suitable sources for optimizing both growth and antimicrobial activity for each strain. Inorganic nitrogen sources alone were insufficient to support growth in the tested strains, and no antibacterial activity was detected under these conditions, reinforcing the necessity of organic nitrogen sources. Additionally, nitrogen sources obtained through acid hydrolysis proved ineffective in promoting antimicrobial activity, likely due to the degradation of essential components during the hydrolysis process. Despite the inherent challenges of transitioning from animal-derived to alternative nitrogen sources in culture media, this study demonstrates the feasibility of such a shift. Plant-based nitrogen sources, including those derived from soy and wheat, along with microbial sources such as yeast, effectively supported the growth and antibacterial activity of the LL, WC, and PA strains, respectively.

This research contributes to the optimization of fermentation processes for postbiotic production while addressing key challenges in food microbiology and sustainability. By exploring animal-free culture media, this study supports the growing demand for more sustainable and ethically produced food systems. Integrating these approaches into food production could foster environmentally friendly practices while preserving the functional integrity of food products.

Future studies should explore the critical components of the growth medium and their optimal concentrations to better understand their impact on bacterial growth and antimicrobial activity. Investigating the specific interactions between different carbon and nitrogen sources could provide valuable insights into tailoring cultivation strategies for specific lactic acid bacteria (LAB) strains. Additionally, examining the influence of environmental factors, such as temperature, agitation, and pH, on the production of postbiotics could help optimize yields and improve the overall efficiency of fermentation processes.

## Figures and Tables

**Table 1:** Effect of different carbon sources on the bacterial concentration and pH values of three strains after 24 h of incubation at 37 °C.

Carbon Source	PA		WC		LL	
	Log CFU/mL	pH	Log CFU/mL	pH	Log CFU/mL	pH
Fructose	10.17 ± 0.21 ^A^	3.67 ± 0.02 ^E^	9.39 ± 0.16 ^A^	4.20 ± 0.02 ^H^	7.74 ± 0.10 ^C^	4.47 ± 0.01 ^E^
Maltose	9.07 ± 0.12 ^C^	6.06 ± 0.01 ^A^	7.74 ± 0.07 ^D^	4.17 ± 0.01 ^H^	8.46 ± 0.10 ^B^	4.65 ± 0.01 ^C^
Xylose	9.97 ± 0.23 ^AB^	3.95 ± 0.01 ^D^	7.73 ± 0.19 ^D^	4.48 ± 0.01 ^F^	9.37 ± 0.22 ^A^	4.44 ± 0.01 ^E^
Ribose	10.07 ± 0.21 ^AB^	4.03 ± 0.06 ^C^	8.18 ± 0.04 ^C^	4.82 ± 0.02 ^E^	8.44 ± 0.01 ^B^	4.71 ± 0.01 ^B^
Trehalose	9.93 ± 0.15 ^AB^	3.67 ± 0.01 ^E^	8.52 ± 0.16 ^BC^	5.05 ± 0.02 ^D^	8.45 ± 0.09 ^B^	4.44 ± 0.01 ^E^
Lactose	9.87 ± 0.06 ^AB^	5.80 ± 0.01 ^B^	8.59 ± 0.08 ^BC^	6.63 ± 0.02 ^A^	8.45 ± 0.21 ^B^	4.56 ± 0.01 ^D^
Galactose	9.63 ± 0.25 ^B^	3.91 ± 0.01 ^D^	8.65 ± 0.11 ^B^	5.48 ± 0.02 ^C^	8.66 ± 0.20 ^B^	4.73 ± 0.01 ^B^
Starch	9.60 ± 0.20 ^B^	5.75 ± 0.02 ^B^	8.79 ± 0.18 ^B^	5.97 ± 0.00 ^B^	9.32 ± 0.04 ^A^	6.40 ± 0.02 ^A^
Glucose	9.83 ± 0.07 ^AB^	3.69 ± 0.01 ^E^	8.67 ± 0.23 ^B^	4.28 ± 0.01 ^G^	8.40 ± 0.27 ^B^	4.18 ± 0.01 ^F^

*Pediococcus acidilactici* (PA), *Weissella cibaria* (WC), and *Lactococcus lactis* (LL). Results are expressed as means ± SDs. Means in the same column with different uppercase letters are significantly different (*p* ≤ 0.05).

**Table 2:** Effect of different nitrogen sources of animal origin on the bacterial concentration and pH values of three strains after 24 h of incubation at 37 °C.

Animal Nitrogen Source	PA		WC		LL	
	Log CFU/mL	pH	Log CFU/mL	pH	Log CFU/mL	pH
Acid casein peptone	7.53 ± 0.23 ^D^	6.55 ± 0.02 ^A^	7.25 ± 0.20 ^E^	6.47 ± 0.02 ^A^	7.13 ± 0.05 ^B^	5.91 ± 0.01 ^A^
Casein peptone	9.80 ± 0.00 ^B^	4.12 ± 0.02 ^E^	7.86 ± 0.05 ^D^	4.10 ± 0.02 ^G^	8.12 ± 0.02 ^A^	4.30 ± 0.02 ^D^
Proteose peptone No.3	10.17 ± 0.06 ^AB^	3.72 ± 0.03 ^I^	7.77 ± 0.06 ^D^	4.08 ± 0.01 ^GH^	8.31 ± 0.19 ^A^	4.44 ± 0.02 ^C^
Peptonized milk	8.33 ± 0.15 ^C^	6.02 ± 0.02 ^B^	9.00 ± 0.17 ^AB^	4.69 ± 0.02 ^C^	8.71 ± 0.10 ^A^	4.12 ± 0.02 ^H^
Meat extract	10.07 ± 0.15 ^AB^	3.72 ± 0.01 ^I^	8.23 ± 0.25 ^CD^	3.99 ± 0.02 ^I^	8.71 ± 0.06 ^A^	4.18 ± 0.01 ^G^
Tryptone	10.30 ± 0.17 ^A^	3.94 ± 0.02 ^FG^	9.30 ± 0.17 ^A^	4.06 ± 0.02 ^GH^	8.28 ± 0.23 ^A^	4.28 ± 0.02 ^DE^
Gelatin peptone	10.13 ± 0.15 ^AB^	4.30 ± 0.01 ^D^	9.20 ± 0.10 ^A^	4.00 ± 0.02 ^I^	8.37 ± 0.14 ^A^	4.19 ± 0.02 ^FG^
Bacteriological peptone	10.10 ± 0.27 ^AB^	4.29 ± 0.02 ^D^	9.33 ± 0.15 ^A^	4.05 ± 0.02 ^H^	8.37 ± 0.29 ^A^	4.23 ± 0.01 ^EF^
Lactalbumin hydrolysate	10.30 ± 0.10 ^A^	3.79 ± 0.01 ^H^	7.77 ± 0.06 ^D^	4.25 ± 0.01 ^EF^	8.31 ± 0.30 ^A^	4.21 ± 0.01 ^FG^
Acid casein hydrolysate	7.43 ± 0.12 ^D^	5.68 ± 0.00 ^A^	7.26 ± 0.26 ^E^	6.07 ± 0.02 ^B^	6.23 ± 0.24 ^C^	5.68 ± 0.02 ^B^
Enzymatic casein hydrolysate	9.93 ± 0.06 ^AB^	3.89 ± 0.02 ^G^	7.78 ± 0.06 ^D^	4.28 ± 0.01 ^E^	8.27 ± 0.27 ^A^	4.28 ± 0.02 ^D^
Bacteriological peptone type II	10.03 ± 0.06 ^AB^	3.63 ± 0.02 ^J^	7.99 ± 0.14 ^D^	4.22 ± 0.01 ^F^	8.20 ± 0.08 ^A^	4.20 ± 0.03 ^FG^
Casein peptone type IV	9.87 ± 0.15 ^B^	3.96 ± 0.01 ^F^	8.23 ± 0.23 ^CD^	4.36 ± 0.01 ^D^	8.37 ± 0.25 ^A^	4.17 ± 0.01 ^G^
MRS (control)	9.83 ± 0.07 ^B^	3.69 ± 0.01 ^I^	8.67 ± 0.23 ^BC^	4.28 ± 0.01 ^E^	8.40 ± 0.27 ^A^	4.18 ± 0.01 ^FG^

*Pediococcus acidilactici* (PA), *Weissella cibaria* (WC), and *Lactococcus lactis* (LL). Results are expressed as means ± SDs. Means in the same column with different uppercase letters are significantly different (*p* ≤ 0.05).

**Table 3:** Effect of different nitrogen sources of non-animal origin on the bacterial concentration and pH values of three strains after 24 h of incubation at 37 °C.

Non-Animal Nitrogen Source	PA		WC		LL	
	Log CFU/mL	pH	Log CFU/mL	pH	Log CFU/mL	pH
Pea hydrolysate 1.1	9.83 ± 0.06 ^ABC^	5.36 ± 0.02 ^B^	8.63 ± 0.29 ^CDE^	5.67 ± 0.01 ^B^	8.60 ± 0.10 ^ABC^	4.36 ± 0.01 ^D^
Rice hydrolysate	8.53 ± 0.15 ^G^	6.44 ± 0.01 ^A^	7.97 ± 0.06 ^G^	6.45 ± 0.02 ^A^	8.87 ± 0.21 ^AB^	4.93 ± 0.02 ^B^
Soy peptone	10.03 ± 0.06 ^A^	3.80 ± 0.01 ^H^	8.00 ± 0.30 ^FG^	3.97 ± 0.02 ^K^	8.93 ± 0.12 ^A^	4.22 ± 0.02 ^FG^
Wheat peptone	9.93 ± 0.06 ^AB^	4.04 ± 0.03 ^G^	9.27 ± 0.06 ^AB^	4.30 ± 0.03 ^G^	8.51 ± 0.19 ^ABC^	4.39 ± 0.02 ^D^
Malt extract	9.00 ± 0.17 ^EF^	4.86 ± 0.01 ^D^	9.20 ± 0.20 ^AB^	4.64 ± 0.01 ^D^	8.10 ± 0.10 ^CD^	4.25 ± 0.02 ^F^
Acid soy hydrolysate	7.23 ± 0.15 ^H^	5.36 ± 0.01 ^B^	7.43 ± 0.06 ^H^	5.66 ± 0.02 ^B^	4.63 ± 0.25 ^E^	5.60 ± 0.02 ^A^
Enzymatic soy hydrolysate	9.73 ± 0.12 ^ABC^	3.53 ± 0.01 ^L^	7.33 ± 0.21 ^H^	4.28 ± 0.01 ^G^	8.60 ± 0.17 ^ABC^	4.02 ± 0.01 ^K^
Ultra-filtered cotton hydrolysate	9.87 ± 0.06 ^ABC^	3.72 ± 0.01 ^IJ^	8.53 ± 0.06 ^DE^	4.21 ± 0.01 ^H^	8.40 ± 0.27 ^BCD^	4.13 ± 0.01 ^IJ^
Soy peptone type III	9.30 ± 0.20 ^DE^	3.79 ± 0.03 ^H^	8.80 ± 0.17 ^BCD^	4.31 ± 0.01 ^G^	8.57 ± 0.12 ^ABC^	4.13 ± 0.02 ^IJ^
Pea hydrolysate 3.0	9.30 ± 0.20 ^DE^	5.03 ± 0.02 ^C^	8.57 ± 0.06 ^DE^	4.76 ± 0.03 ^C^	8.27 ± 0.12 ^CD^	4.39 ± 0.02 ^D^
Ultra-filtered wheat hydrolysate	9.77 ± 0.06 ^ABC^	4.25 ± 0.02 ^F^	9.40 ± 0.17 ^A^	4.12 ± 0.01 ^I^	8.60 ± 0.10 ^ABC^	4.16 ± 0.02 ^HI^
Ultra-filtered rice hydrolysate	9.57 ± 0.15 ^BCD^	4.49 ± 0.02 ^E^	9.10 ± 0.20 ^ABC^	4.47 ± 0.01 ^E^	8.57 ± 0.15 ^ABC^	4.31 ± 0.02 ^E^
Yeast extract 5.4	9.47 ± 0.23 ^CD^	3.74 ± 0.03 ^I^	8.50 ± 0.20 ^DEF^	4.02 ± 0.01 ^J^	8.51 ± 0.01 ^ABC^	4.52 ± 0.02 ^C^
Yeast extract 1.1	8.87 ± 0.12 ^FG^	4.84 ± 0.02 ^D^	8.60 ± 0.10 ^CDE^	4.22 ± 0.03 ^H^	8.53 ± 0.15 ^ABC^	4.04 ± 0.01 ^K^
Yeast extract 5.5	9.57 ± 0.12 ^BCD^	3.58 ± 0.01 ^K^	8.17 ± 0.12 ^EFG^	4.37 ± 0.02 ^F^	8.00 ± 0.10 ^D^	4.10 ± 0.01 ^J^
MRS (control)	9.83 ± 0.07 ^ABC^	3.69 ± 0.01 ^J^	8.67 ± 0.23 ^CDE^	4.28 ± 0.01 ^G^	8.40 ± 0.27 ^BCD^	4.18 ± 0.01 ^GH^

*Pediococcus acidilactici* (PA), *Weissella cibaria* (WC), and *Lactococcus lactis* (LL). Results are expressed as means ± SDs. Means in the same column with different uppercase letters are significantly different (*p* ≤ 0.05).

**Table 4:** Effect of different inorganic sources on the bacterial concentration and pH values of three strains after 24 h of incubation at 37 °C.

Inorganic Source	PA		WC		LL	
	Log CFU/mL	pH	Log CFU/mL	pH	Log CFU/mL	pH
Ammonium sulfate	7.00 ± 0.14 ^B^	5.90 ± 0.01 ^C^	6.73 ± 0.19 ^BC^	6.48 ± 0.01 ^B^	7.38 ± 0.13 ^B^	6.26 ± 0.01 ^A^
Ammonium chloride	6.45 ± 0.21 ^C^	6.47 ± 0.02 ^B^	6.36 ± 0.13 ^C^	6.41 ± 0.02 ^C^	6.50 ± 0.03 ^C^	6.25 ± 0.03 ^A^
Ammonium citrate	6.39 ± 0.28 ^C^	5.75 ± 0.02 ^D^	6.61 ± 0.14 ^BC^	6.36 ± 0.01 ^D^	4.55 ± 0.16 ^D^	6.31 ± 0.08 ^A^
Potassium nitrate	0.00 ± 0.00 ^E^	NM	6.84 ± 0.17 ^B^	6.57 ± 0.02 ^A^	0.00 ± 0.00 ^E^	NM
Sodium nitrate	4.62 ± 0.22 ^D^	6.53 ± 0.01 ^A^	6.67 ± 0.23 ^BC^	6.50 ± 0.01 ^B^	0.00 ± 0.00 ^E^	NM
Sodium citrate	0.00 ± 0.00 ^E^	NM	6.54 ± 0.11 ^BC^	6.46 ± 0.02 ^B^	0.00 ± 0.00 ^E^	NM
Sodium sulphate	0.00 ± 0.00 ^E^	NM	5.49 ± 0.17 ^D^	6.54 ± 0.02 ^A^	0.00 ± 0.00 ^E^	NM
MRS (control)	9.38 ± 0.07 ^A^	3.69 ± 0.01 ^E^	8.67 ± 0.23 ^A^	4.28 ± 0.01 ^E^	8.40 ± 0.00 ^A^	4.18 ± 0.01 ^B^

NM = Not Measured. *Pediococcus acidilactici* (PA), *Weissella cibaria* (WC), and *Lactococcus lactis* (LL). Results are expressed as means ± SDs. Means in the same column with different uppercase letters are significantly different (*p* ≤ 0.05).

## Data Availability

The original contributions presented in this study are included in the article. Further inquiries can be directed to the corresponding author.

## Abbreviations

The following abbreviations are used in this manuscript:

PA	*Pediococcus acidilactici* CECT 9879
WC	*Weissella cibaria* CECT 30731
LL	*Lactococcus lactis* CECT 30734
